# Rational Design of Gene Therapy Vectors

**DOI:** 10.1016/j.omtm.2019.01.009

**Published:** 2019-02-16

**Authors:** Dao Pan, Hildegard Büning, Chen Ling

**Affiliations:** 1 Division of Experimental Hematology and Cancer Biology, Cincinnati Children’s Hospital Medical Center, Cincinnati, OH, USA; 2 Department of Pediatrics, University of Cincinnati, Cincinnati, OH, USA; 3 Institute of Experimental Hematology, Hannover Medical School, Hannover, Germany; 4 REBIRTH Cluster of Excellence, Hannover Medical School, Hannover, Germany; 5 Division of Cellular and Molecular Therapy, Department of Pediatrics, University of Florida, Gainesville, FL, USA; 6 State Key Laboratory of Genetic Engineering, School of Life Sciences, Zhongshan Hospital, Fudan University, Shanghai, China

## Main Text

Gene therapy has entered clinical reality with marketing authorizations in Europe and the US for the treatment of patients with inherited or acquired diseases, including inborn blindness, adenosine deaminase deficiency, and certain types of cancer.^1^ Appropriate vector design is pivotal to the development of successful gene therapy, which often requires robust and sustained transgene expression in properly targeted cells while avoiding transgene-related adverse effects (such as off-target or overexpression-associated cytotoxicity or immune responses). In this special issue of *Molecular Therapy - Methods & Clinical Development*, which is on “Rational Design of Gene Therapy Vectors,” seven review articles highlight current efforts to improve the efficacy of gene therapy. Delivery tools and transgene expression cassettes are being designed and optimized for the treatment of diseases ranging from rare genetic disorders to major public health concerns, such as cancer.

For instance, lentiviral vectors (LVs) serve as powerful gene delivery tools, with well-documented success in *ex vivo* hematopoietic stem cell (HSC)-based gene therapy. Thus, LVs with targeted delivery and/or transgene expression are being developed. Frank and Buchholz^2^ review various strategies to engineer envelope glycoproteins toward targeting LV gene delivery to distinct subtypes of lymphocytes for the treatment of inherited immunodeficient diseases and for cancer immunotherapy. To generate such receptor-specific LVs, three steps must be accomplished simultaneously: destroying natural receptor binding, targeting new ligands for cell binding, and modifying viral glycoproteins for cell entry. Various LV envelope proteins, targeted ligands, and their receptors are discussed in detail, followed by a summary of current working models on cell membrane fusion, cell entry, and intracellular trafficking. Because of current limitations of LVs, lymphocyte gene transfer predominately relies on *ex vivo* transduction, which produces challenges on effective *ex vivo* cultivation and engraftment. Given recent advances in the development of targeted LV vectors, future approaches may allow for efficient *in vivo* gene delivery and transduction. For example, *in vivo* reprogramming of chimeric antigen receptor (CAR)-T cells has recently been reported using a CD8-targeted LV. In addition to engineering LV tropism(s) toward desired cell types, the design of the transgene expression cassette is of substantial importance in achieving targeted expression. Merlin and Follenzi^3^ review the application of various regulatory elements at both transcriptional and post-transcriptional levels for restriction of transgene expression in LV-mediated *ex vivo* and *in vivo* gene therapy approaches. Three main strategies are discussed: the restriction of transduction by controlling LV tropism for the desired cell types, the use of cell-type-specific promoters (either naturally present or hybrid and/or synthetic) for targeted expression, and microRNA-dependent post-transcriptional regulation for de-targeting expression from “off-target” cell types (especially antigen-presenting cells). Particular emphasis is given to the design of LV transfer expression cassettes for use in gene therapy of bleeding disorders, X-linked chronic granulomatous disease, Wiscott-Aldrich syndrome, Alzheimer’s disease, as well as various cancers.

Büning and Srivastava^4^ focus on adeno-associated viral (AAV) vectors, the most frequently used viral vectors for *in vivo* gene therapy, and summarize recent advances in the engineering of the viral capsid to improve vector specificity and/or efficiency. For example, through the insertion of receptor-binding ligands into surface exposed positions of the capsid, cell types that are non-permissive or low permissive for natural AAV serotypes become susceptible to transduction with AAV vectors. In addition, vector tropism can be re-directed toward a target cell of choice by combining insertion of a ligand with site-directed mutagenesis to destroy the capsid’s natural receptor binding motifs. To illustrate how capsid engineering can be applied to impact AAV’s intracellular fate, strategies to protect the viral capsid against recognition by the host cell proteasomal degradation machinery are presented. Using such vectors, *ex vivo* and *in vivo* transduction efficiencies were significantly improved.

Not every strategy for gene delivery and expression can be universally applied. For instance, hemoglobinopathies represent unique treatment challenges that require both targeted and controlled expression in the erythroid lineage, while needing high levels to achieve therapeutic benefits. Davis et al.^5^ provide an overview of current gene engineering approaches, with a particular emphasis on *ex vivo* HSC-based gene transfer and gene editing in combination with autologous transplantation. The authors describe the complexities of the diseases that are associated with either a single mutation in the adult β-globin gene in sickle cell disease or an imbalance of globin chain production in thalassemia major. Moreover, multiple genes are targeted in diverse approaches, including α-globin, adult wild-type or mutated β-globin, and fetal γ-globin. Ongoing globin gene therapy trials primarily rely on traditional overexpression approaches (gene addition strategies), which require the optimal choice of promoters, DNA regulatory elements, and insulators. Promising alternative strategies include gene editing approaches to either reduce α-globin expression or increase (or reactivate) fetal γ-globin gene by targeting endogenous regulatory elements of globin or repressor genes. Samelson-Jones and Arruda^6^ further elaborate on transgene engineering using treatment of hemophilia as an example. Bioengineering strategies for coagulation factor VIII (hemophilia A) and factor IX (hemophilia B) to enable vectorization, improve functionality, and lower the risk of immune responses are discussed in detail. Efficacy of hemophilia gene therapy with regard to coagulation factor expression levels is impressive as supranormal levels are now achieved. Such high efficacy is unusual in traditional gene transfer strategies and therefore raises the question of whether a fine tuning of transgene expression is needed, as supranormal levels of coagulation factors might increase thrombotic risk. It should be pointed out that antibody formation against systemically delivered transgene products is a key challenge in hemophilia and other replacement therapies that also needs to be considered during bioengineering of proteins.

Besides viral vectors, non-viral or synthetic vectors are being developed for gene therapy. In their review, Xiao et al.^7^ report the state-of-the-art bioengineering of synthetic nanoparticles, with a particular emphasis on targeted *in vivo* delivery to solid tumors. Generally, synthetic nanoparticles are lipid-based, polymeric, or based on inorganic materials. Initially designed merely as a coat to protect the genetic payload and to shield its negative charge, non-viral RNA or DNA carriers are now carefully engineered to target distinct cell surface molecules and entry routes. In addition, knowledge of the tumor-specific microenvironment is efficiently explored as a means to convey tumor-selective gene delivery. Furthermore, switches, such as light response systems, are in development to fine-tune target cell transduction. Owing to these technical advances, as well as reduced immunogenicity and production costs, synthetic nanoparticles have emerged as potent alternatives to viral vectors as delivery vehicles for gene therapy. Alternatively, effective cell therapy-based approaches to cancer treatment have been established in recent years. CAR-T cells are revolutionizing the field of cancer immunotherapy. Design of the CAR is paramount to achieve specific targeting of antigens associated with cancer cells and optimal activation of the engineered T cell. Guedan et al.^8^ explain this complex topic by first summarizing each of the modules that form a CAR: extracellular antigen-binding domain, hinge region, transmembrane domain, and costimulatory domain. When properly assembled, CARs can successfully activate T cells and redirect them in an antigen-specific fashion. Next, the authors provide a comprehensive overview of strategies to generate CAR-T cells: viral gene transfer, non-viral DNA transfection, and, more recently, genome editing. The authors also draw attention to the major challenges for CAR-T cell therapy, such as tumor escape, tonic signaling, off-tumor toxicities, and inefficiency of targeting solid tumors. In this regard, new approaches fueled by rapid advances in synthetic biology are introduced to produce the next generation of CARs and overcome these challenges. In the end, an innovative versatile strategy, the utilization of “universal CARs” that allow targeting of multiple tumor antigens, is discussed. In conclusion, rational designs by molecular engineering wizards continue to generate ever more potent and more targeted vectors and cells as well as tools for gene expression, regulation, and editing. These molecular approaches are an integral component of the current revolution and success of molecular medicine.
